# Quantifying Gait Impairment Using an Instrumented Treadmill in People with Multiple Sclerosis

**DOI:** 10.1155/2013/867575

**Published:** 2013-06-25

**Authors:** Alon Kalron, Zeevi Dvir, Lior Frid, Anat Achiron

**Affiliations:** ^1^Multiple Sclerosis Center, Sheba Medical Center, Tel Hashomer, Israel; ^2^Institute of Motor Functions, Sheba Medical Center, Tel Hashomer, Israel; ^3^Sackler Faculty of Medicine, Tel-Aviv University, Israel

## Abstract

*Background and Objective*. Treadmill gait analysis has been proposed as an attractive alternative for overground walking measuring systems. The purpose of this study was twofold: first to determine spatiotemporal parameters of treadmill gait in patients with multiple sclerosis (MS) and second to examine whether these parameters are associated with specific functional impairments in this cohort. *Method*. Eighty-seven relapsing-remitting patients diagnosed with MS, 50 women and 37 men, aged 40.9 ± 11.9 with an expanded disability status scale (EDSS) score of 2.7 ± 1.6, participated in this study. Twenty-five apparently healthy subjects, 14 women and 11 men, aged 38.5 ± 9.4, served as controls. Spatiotemporal gait parameters were obtained using the Zebris FDM-T Treadmill (Zebris Medical GmbH, Germany). People with MS demonstrated significantly shorter steps, extended stride time, wider base of support, longer step time, reduced single support phase, and a prolonged double support phase compared to the healthy controls. The EDSS score was significantly correlated with all spatiotemporal gait parameters. *Conclusion*. The instrumented treadmill may be an effective tool in assessing ambulation capabilities of people with MS.

## 1. Introduction

Multiple sclerosis (MS) is a neurologic disease affecting an estimated 2.5 million adults worldwide and is the most common disabling neurological disease in young adults. MS is diagnosed between ages 20 and 50 [1], is 2 to 3 times more common in women than in men [2], and results in demyelination and axonal loss in the central nervous system (CNS) [3]. Such CNS damage may result in ambulatory limitations which are key components of disability in patients with MS [4]. Approximately 75% of these patients experience clinically significant walking and balance disturbances [5] which may present even in the early stages of the disease and in individuals diagnosed with clinically isolated syndrome [6].

 Gait impairments are associated with the severity of deficiency in various functional neurological systems, that is, muscle power loss, level of spasticity, degree of instability due to impaired coordination, and degree of sensory impairment. Impairment in one functional system alone or in combination contributes to the patient's gait impairment, resulting in a specific gait pattern [7]. Variations may also be seen in the same patient over a period of time [8]. Therefore, increasing awareness of walking and balance limitations in this patient group requires continuous evaluation. Assessment is essential in order to monitor disease dynamics and assess the efficacy of symptomatic and rehabilitation therapies. 

 Assessment of gait performance in people with MS consists of clinician-assessed rating scales, performance tests, and self-reporting questionnaires. With the advent of modern instrumented walkway systems, basic spatiotemporal gait parameters have been increasingly used by clinicians to define the characteristics of pathological gait due to MS and to assess interventions aimed at improving gait. It is widely accepted that people with MS walk at a slower speed, extend the period of time when both legs contact the ground, and have a wider base of support compared to healthy subjects [9, 10]. Moreover, Givon et al. (2009) demonstrated significant correlations between various spatiotemporal gait parameters with the level of neurological impairment due to MS [9]. 

 On the other hand, length restrictions of commercial instrumented walkways render them suboptimal for investigating long-distance locomotion. Moreover, since the standard electronic walkways analyze only a small number of steps, it may not reflect the impact of fatigue on gait performance.

 Recently, treadmill gait analysis has been proposed as an attractive alternative for overground walking measuring systems [11]. The instrumented treadmill enables collection of gait performance over long distances and/or long periods of time using standardized conditions, namely, distance, belt speed, and inclination. Treadmills require less space and in specific models, can be safely operated due to the overhanging harness which is equipped with an emergency belt stop option if the subject/patient trips or collapses. 

 Although instrumented treadmills have generated outcome measures relating to acquired brain injury [12], no study to date has evaluated gait with instrumented treadmills in people with MS. Therefore, the purpose of this study was twofold: first to determine spatiotemporal parameters of treadmill gait in patients with MS and second to examine whether these parameters are associated with specific functional impairments in this cohort.

## 2. Methods 

### 2.1. Study Participants

This is an observational case control study. Eighty-seven relapsing-remitting patients diagnosed with MS, 50 women and 37 men, aged 40.9 ± 11.9 , were recruited from the Multiple Sclerosis Center, the Sheba Medical Center, Tel-Hashomer, Israel, and participated in this investigation. Inclusion criteria included (1) a neurologist-confirmed diagnosis of definite relapsing-remitting MS according to the revised McDonald criteria [13]; (2) less than 6 on the expanded disability status scale (EDSS), equivalent to walking without an assistive device (e.g., a cane or walker); and (3) relapse-free for at least 30 days prior to testing. Exclusion criteria included (1) orthopedic disorders that could negatively affect mobility; (2) major depression or cognitive decline; (3) pregnancy; (4) blurred vision; and (5) cardiovascular disorders. Twenty-five apparently healthy subjects, 14 women and 11 men, aged 38.5 ± 9.4, served as controls. The study was approved by the Sheba Institutional Review. All participating subjects signed an informed consent form.

### 2.2. Gait Analysis

Gait spatiotemporal parameters were obtained using the Zebris FDM-T Treadmill (Zebris Medical GmbH, Germany). The Zebris FDM-T is fitted with an electronic mat of 10,240 miniature force sensors, each approximately 0.85 × 0.85 cm embedded underneath the belt. The treadmill's contact surface measures 150 × 50 cm and its speed can be adjusted from 0.2 to 22 km/h, at intervals of 0.1 km/h. When the subject stands/walks on the treadmill, the force exerted by his feet (the so-called reactive-normal force) is recorded by the sensors at a sampling rate of 120 Hz. Due to the high density of the sensors, the foot is mapped at a high resolution to facilitate even subtle changes in force distribution. Timing can also be monitored. Dedicated software integrates the force signals and provides 2D/3D graphic representation of major spatiotemporal parameters including center of pressure (CoP) trajectories during static stance and gait. 

**Figure 1:**
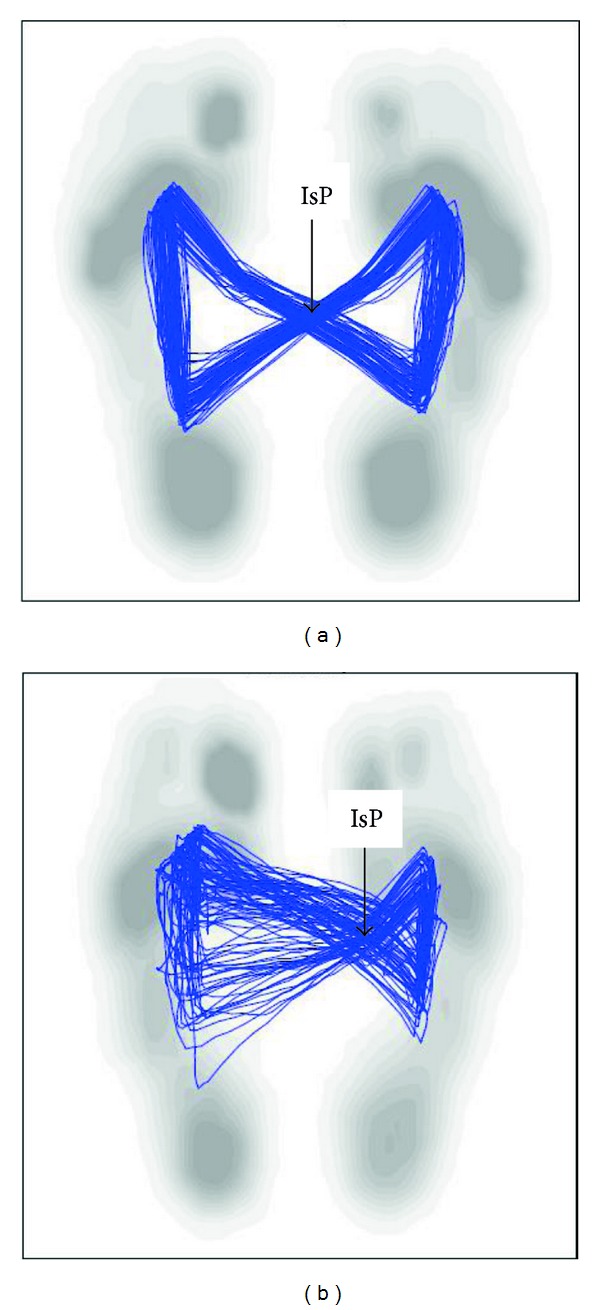
Cyclogram (butterfly) diagrams derived from the instrumented treadmill: (a) healthy participant, (b) person with MS (EDSS = 4.5). IsP, intersection point.

Faude et al. (2012) reported high levels of between- and within-day reliability in healthy seniors for the majority of spatiotemporal gait parameters recorded by the Zebris treadmill system during walking, with coefficients of variation typically below 5% and 7%, respectively [11]. The dedicated software also generates a graphic pattern termed the “butterfly”, representing a continuous trace of the CoP trajectory during walking (Figure 1). 

 The following set of parameters was derived from the butterfly.

1. Anterior/posterior variability (mm): defined as the standard deviation of the intersection point in the anterior/posterior direction.
2. Lateral symmetry (mm): left/right shift of the intersection point; “zero position” is equivalent to perfect symmetry.
3. Lateral variability (mm): defined as the standard deviation of the lateral symmetry.

Prior to the measurement phase, all participants actively participated in an adaptation-familiarization trial in order to establish each individual's speed level. Starting at a fixed speed of 0.5 km/h, the belt speed was increased by 0.3 km/h every 15 seconds in a stepwise manner. Once the participant informed the tester that speed that best characterized his/her normal walking pace was determined as his/her comfort speed, following this adaptation phase, each participant was instructed to walk without shoes on the treadmill for one consecutive minute at the comfort speed. 

### 2.3. Statistical Analysis

Group differences in age and gender distribution were determined using an independent sample *t*-test and chi-square test, respectively. All spatiotemporal gait data were normally distributed and did not violate homogeneity of variance. Differences in average and variability of gait parameters between individuals with MS and controls were determined using an independent sample *t*-test. The magnitudes of group differences were indexed by a 95% confidence interval (95% CI). Spearman's rank correlation coefficient was used to examine the association between spatiotemporal gait parameters and level of neurological impairment defined by the EDSS. All analyses were performed using SPSS Version 21.0 SPSS Inc., NY, USA). All reported *P* values were two-tailed. The level of significance was set at *P* < 0.05.

## 3. Results

The patient group had a mean duration of 6.0 ± 6.2 years since diagnosis and an EDSS score of 2.7 ± 1.6, representing moderate neurological disability. The mean pyramidal, cerebellar, and sensory scores were 1.6 ± 1.2, 1.0 ± 1.1, and 0.8 ± 1.0, respectively. Other participants' related clinical scores are outlined in Table 1.

Table 2 refers to the spatiotemporal gait parameters during treadmill walking. Significant differences were observed between patients and healthy subjects with respect to all 14 parameters. People with MS demonstrated shorter steps, extended stride time, and a wider base of support compared to the healthy controls. Patients walked with a longer step time, reduced single support phase, and a longer period of time with both legs on the treadmill's belt (double support). It is worth noting that although both groups were instructed to increase the treadmill speed to comfort level, the patients' self-selected velocity was 59% slower than their healthy counterparts (2.2 versus 3.5 km/h, *P* < 0.001). 

 With respect to the butterfly diagram, significant differences between groups were observed in the lateral symmetry, anterior-posterior variability, and lateral variability parameters. Patients scored on average 2.5 times higher in all three parameters compared to the healthy participants. 

 The EDSS correlated significantly with the mean score of all spatiotemporal parameters. The strongest correlation was with the double support parameter (Spearman's rho = 0.65). Moderate associations were found during the single support period and stance phase: rho −0.61, 0.61 (Spearman's rho), respectively. The least correlated were step, stride time and width of support: 0.28, 0.31, 0.35  (rho), respectively.

 Positive correlations were observed between the EDSS score and parameters derived from the butterfly diagram. Of the three parameters, the anterior-posterior and lateral variability parameters revealed the strongest correlations: Spearman's rho = 0.55, 0.56 (rho), respectively. The correlation scores between spatiotemporal gait parameters and the neurological variables are outlined in Table 3.

## 4. Discussion

Our primary aim was to examine if spatiotemporal parameters derived from an instrumented treadmill (Zebris Medical GmbH) could distinguish between people with MS and age, gender, height and weight matched controls. Main spatiotemporal gait features demonstrated by people with MS were a slower self-selected speed, shorter step and stride length, wider base of support and a prolonged double support phase, compared to the healthy participants. Our findings are consistent with those previously presented in relatively large groups of MS patients, however, performed on an electronic walkway [9, 10]. Since treadmill gait analysis is a relatively new measurement device for gait analysis in people with MS, it is worth discussing the difference between the current study gait instrument to conventional over ground gait measurement tools. 

 Walking on an instrumented treadmill, used in this study, has several advantages compared to an over ground device with similar footprint analysis capability. Specifically, compared to the GAITRite electronic walkway, treadmill gait analysis is not limited to the number of steps per trial. This advantage enables gait analysis over large distances. We assume that this ability is essential in the MS population due to fatigue concerns. Recently, energy cost of walking was significantly and inversely associated with gait speed (*r* = −.25) and stride length (*r* = −.32) and positively associated with double limb support (*r* = .27) in people with mild MS [14]. Secondly, with an instrumented treadmill the individual is able to control the speed, allowing for more standardized testing of gait characteristics including the high end of gait jogging. Thirdly, this innovative device enables gait measurement while walking on an inclined surface, a condition that may correspond to several active daily situations (e.g., walking up hill or on sloped roads). Furthermore, we believe that the instrumented treadmill is better fitted for measuring gait performance in studies involving an intervention program based on treadmill training, body weight treadmill training and robot-assisted treadmill training. 

 On the other hand, treadmill gait analysis poses other problems as it may impede natural walking patterns. Indeed, some parameters (e.g., cadence, stance period) are modified. Energy costs have been reported to be higher during treadmill walking than ground walking [15] while kinematic parameters during both tasks are inconsistent [15, 16]. Moreover, evidence suggests that due to the automatic and regular drive to the lower limbs, kinesthetic and external afferent impulses may differ between (both) tasks which may affect the generation of locomotor patterns [15]. These aspects should be taken into consideration when interpreting treadmill gait findings and implementation of observed gait deviations in intervention strategies. 

 According to these statements, we believe that the relatively slow-selected speed, decreased step length, and prolonged double support demonstrated by the current patient group were associated with the afferent impulse derived by the treadmill. Normal performance of walking on a treadmill is characterized by a positive correlation between belt speed to step length and pace of steps. That is to say, that if the walking subject fails to increase at least one of these two spatiotemporal variables, as a response to an increase in speed, he is at risk of falling. We assume that, despite the adaptation-familiarization trial, people with MS still felt a lack of confidence while walking on the treadmill. High concerns about falls have been shown to be primarily associated with decreased step lengths in elderly females [17]. We assumed that in order to avoid extended step lengths, people with MS preferred to reduce the treadmill's speed. As a result of the slow speed, short step lengths were maintained and consequently the double support phase increased, which added to balance control. Puh and Baer's study performed on independently ambulatory stroke patients, supports our assumption. Stroke patients demonstrated a significantly slower velocity and longer double support phase in treadmill walking compared to over ground walking [18]. Despite our speculation, future studies focusing on spatiotemporal adaptations in accordance with different speed levels in people with MS are required. 

 A novel contribution of the current system is the butterfly diagram. This diagram, which in a healthy subject resembles a Papillion tie, provides us with information as to the level of symmetry between steps, width of base of support, single and double support phases, and the regularity with which gait is conducted. 

 In the current study, people with MS demonstrated a greater variability in both the sagittal and coronal planes and asymmetrical CoP movement pattern. Interestingly, with the exception of width between steps, these parameters were correlated with the level of neurological disability, for example, the EDSS and the pyramidal and cerebellar functional domains indicated by Spearman's rho ranging 0.55–0.65. Our results are in line with other reports documenting increased variability of spatiotemporal gait parameters in individuals with MS compared to healthy controls [10, 19, 20]. Gait variability has been shown to have clinical import in other special populations, that is, Parkinson, Alzheimers, and so forth and is also associated with falls in the elderly [21]. To date the clinical significance of gait variability in persons with MS is unknown. One investigation [22] demonstrated that recurrent fallers with MS (i.e., 1^+^ falls/year) exhibit greater variability of spatial footfall placement than nonfallers with MS. Nevertheless, following our analysis, indicating a correlation between variability of CoP movement with the EDSS score, we tend to consider gait variability as a relevant marker of mobility deficits in MS. 

 Although persons with MS are clearly characterized with gait impairments, the mechanisms underlying this condition remain unclear. In this regard treadmill gait analysis as performed in the present study had no known added value compared to over ground gait analysis. Based on the belief that gait control involves numerous neural processes and coordination of the trunk and limbs, it is most likely not a single mechanism, but rather a combination of deficits that contribute to a general abnormal gait pattern. Possible factors such as decreased muscle strength [23], spasticity [24], fatigue [25], ataxic movement disorders [26], proprioception, and balance impairments [27] are often cited as contributing mechanisms. There is also some evidence that attention resources are related to gait deficits in persons with MS [28].

 Future studies should take advantage of the instrumented treadmill's specific capabilities, that is, examining gait and balance performance over long distances, information that can potentially improve today's knowledge regarding the impact of fatigue over mobility. Additionally, measurement of walking determinants, while walking on sloped surfaces, can be relevant for minimally impaired MS patients. Moreover, spatiotemporal analysis of high-demanding activities such as jogging or running can be monitored by this device. Finally, a comparison of kinematic and kinetic parameters between treadmills to over ground walking in the MS population is warranted. 

## 5. Conclusion

Our results indicate that the instrumented treadmill is an appropriate tool for assessing ambulation capabilities of people with MS. Furthermore, spatiotemporal gait parameters collected by this device seem to be valid markers of neurological impairment in the MS population. Further study is indicated especially regarding the comparative sensitivity of the treadmill outcome parameters in capturing changes in gait function.

## Figures and Tables

**Table 1:** Demographic, anthropometric, and clinical characteristics of the study population.

Variable	Mean (S.D.)		*P* value
	MS group (*n* = 87)	Healthy subjects (*n* = 25)	
Age (years)	40.9 (11.9)	38.5 (9.4)	0.34
Gender	50 females	14 females	
	37 males	11 males	
Ratio (female/male)	1.35	1.27	0.62
Disease duration (years)	6.0 (6.2)	—	—
Height (cm)	168.3 (8.9)	168.8 (7.9)	0.84
Body mass (kg)	68.8 (13.8)	72.0 (12.4)	0.63
EDSS	2.7 (1.6)	—	—
Pyramidal	1.6 (1.2)	—	—
Cerebellar	1.0 (1.1)	—	—
Sensory	0.8 (1.0)	—	—

EDSS: expanded disability status scale.

**Table 2:** Descriptive statistics of the spatiotemporal gait parameters.

Variable	Mean (S.D.)		Mean difference (95% CI)	*P* value
	MS participants (*n* = 87)	Healthy subjects (*n* = 25)		
Velocity (km/h)	2.2 (1.0)	3.5 (0.7)	−1.3 (−1.7, −0.9)	<0.001
Cadence (steps/min)	94.4 (18.5)	101.1 (11.3)	−6.7 (−12.7, −0.7)	0.03
Step time R (sec)	0.66 (0.15)	0.60 (0.07)	0.06 (0.02, 0.1)	0.007
Step time L (sec)	0.67 (0.15)	0.60 (0.07)	0.07 (0.02, 0.1)	0.002
Step length R (cm)	37.7 (14.8)	57.1 (8.7)	−19.4 (−24.0, −14.7)	<0.001
Step length L (cm)	37.4 (14.5)	57.6 (8.6)	−20.2 (−24.9, −15.6)	<0.001
Stance R (% GC)	69.4 (5.4)	64.4 (2.0)	5.0 (3.6, 6.4)	<0.001
Stance L (% GC)	69.3 (5.6)	64.2 (2.1)	5.1 (3.7, 6.5)	<0.001
Single support R (% GC)	30.6 (5.6)	35.6 (2.1)	−5.0 (−6.6, −3.7)	<0.001
Single support L (% GC)	30.6 (5.5)	35.6 (2.0)	−5.0 (−6.5, −3.6)	<0.001
Double support (% GC)	38.8 (10.5)	28.6 (4.0)	10.2 (7.4, 12.9)	<0.001
Stride time (sec)	1.33 (0.29)	1.20 (0.14)	0.13 (0.04, 0.2)	0.004
Stride length (cm)	75.1 (29.0)	114.7 (17.1)	−39.6 (−51.7, −27.5)	<0.001
Base of support (cm)	13.8 (4.1)	11.0 (3.0)	2.8 (1.1, 4.6)	<0.001

Statistics obtained from the butterfly diagram				
A-P variability (mm)	8.5 (5.9)	3.4 (1.3)	5.1 (3.7, 6.4)	<0.001
Lateral symmetry (mm)	8.6 (7.9)	3.3 (2.5)	5.3 (3.4, 7.3)	0.001
Lateral variability (mm)	18.3 (13.6)	7.6 (8.1)	10.7 (6.4, 15.1)	<0.001

**Table 3:** Bivariate correlations (Spearman's rho) among gait spatiotemporal parameters in persons with MS (*n* = 87).

Treadmill gait variables	EDSS	Pyramidal	Cerebellar
Velocity (km/h)	−0.52*	−0.46*	−0.34*
Cadence (steps/min)	−0.30*	−0.24^∗†^	−0.23*
Step time R (sec)	0.31*	0.22^∗†^	0.29*
Step time L (sec)	0.26*	0.23^∗†^	0.21^∗†^
Step length R (cm)	−0.51*	−0.46*	−0.30*
Step length L (cm)	−0.52*	−0.48*	−0.34*
Stance R (% GC)	0.58*	0.58*	0.38*
Stance L (% GC)	0.63*	0.56*	0.47*
Single support R (% GC)	−0.63*	−0.56*	−0.47*
Single support L (% GC)	−0.58*	−0.58*	−0.38*
Double support (% GC)	0.65*	0.61*	0.44*
Stride time (sec)	0.31*	0.25^∗†^	0.24^∗†^
Stride length (cm)	−0.52*	−0.47*	−0.32*
Base of support (cm)	0.35*	0.29*	NS
A-P variability (mm)	0.55*	0.49*	0.44*
Lateral symmetry (mm)	0.34*	0.42*	0.24^∗†^
Lateral variability (mm)	0.56*	0.50*	0.45*

*Correlation is significant at the 0.01 level (2-tailed). ^∗†^Correlation is significant at the 0.05 level (2-tailed); NS: nonsignificant.
